# A case of progressive multifocal leukoencephalopathy in an adult patient with ADA-SCID

**DOI:** 10.70962/jhi.20250093

**Published:** 2026-07-21

**Authors:** Hannah Shin, Lori Broderick, Jessica Azevedo, Caitlin Mulligan, Kari Hird, Vanessa Goodwill, Courtney Parkin, Stephanie Mortensen, Madisen Traveller, Ahmad Rayes, John Bohnsack, Donald B. Kohn, Teresa K. Tarrant, Michael Hershfield, Jonathan J. Lyons

**Affiliations:** 1 Division of Allergy & Immunology, Department of Medicine, University of California, San Diego, La Jolla, CA, USA; 2 Division of Allergy, Immunology & Rheumatology, Department of Pediatrics, University of California, San Diego Rady Children’s Hospital, San Diego, CA, USA; 3 Department of Neurosciences, University of California, San Diego, La Jolla, CA, USA; 4 Division of Neuropathology, Department of Pathology, University of California, San Diego, La Jolla, CA, USA; 5 Division of Pediatric Hematology/Oncology, Department of Pediatrics, University of Utah, Salt Lake City, UT, USA; 6 Division of Pediatric Rheumatology, Department of Pediatrics, University of Utah, Salt Lake City, UT, USA; 7 Departments of Microbiology, Immunology & Molecular Genetics, David Geffen School of Medicine, University of California, Los Angeles, Los Angeles, CA, USA; 8 Departments of Pediatrics, David Geffen School of Medicine, University of California, Los Angeles, Los Angeles, CA, USA; 9 Division of Rheumatology and Immunology, Department of Medicine, Duke University, Durham, NC, USA; 10 Durham Veterans Affairs, Durham, NC, USA; 11 Veterans Affairs San Diego Healthcare System, La Jolla, CA, USA

## Abstract

This case report describes progressive multifocal leukoencephalopathy in a patient with ADA-SCID status after gene therapy. Persistent metabolic dysfunction likely contributed to JC virus reactivation, highlighting this rare complication of ADA-SCID and incomplete reconstitution seen with early protocols.

## Teaser

This case report describes progressive multifocal leukoencephalopathy in a patient with ADA-SCID who received early gene therapy, highlighting the importance of lifelong monitoring after early gene therapy protocols. Further research in ADA-SCID and advances in gene therapy approaches may improve long-term immune reconstitution and reduce the risk of late complications.

## Case

**Figure 1.**
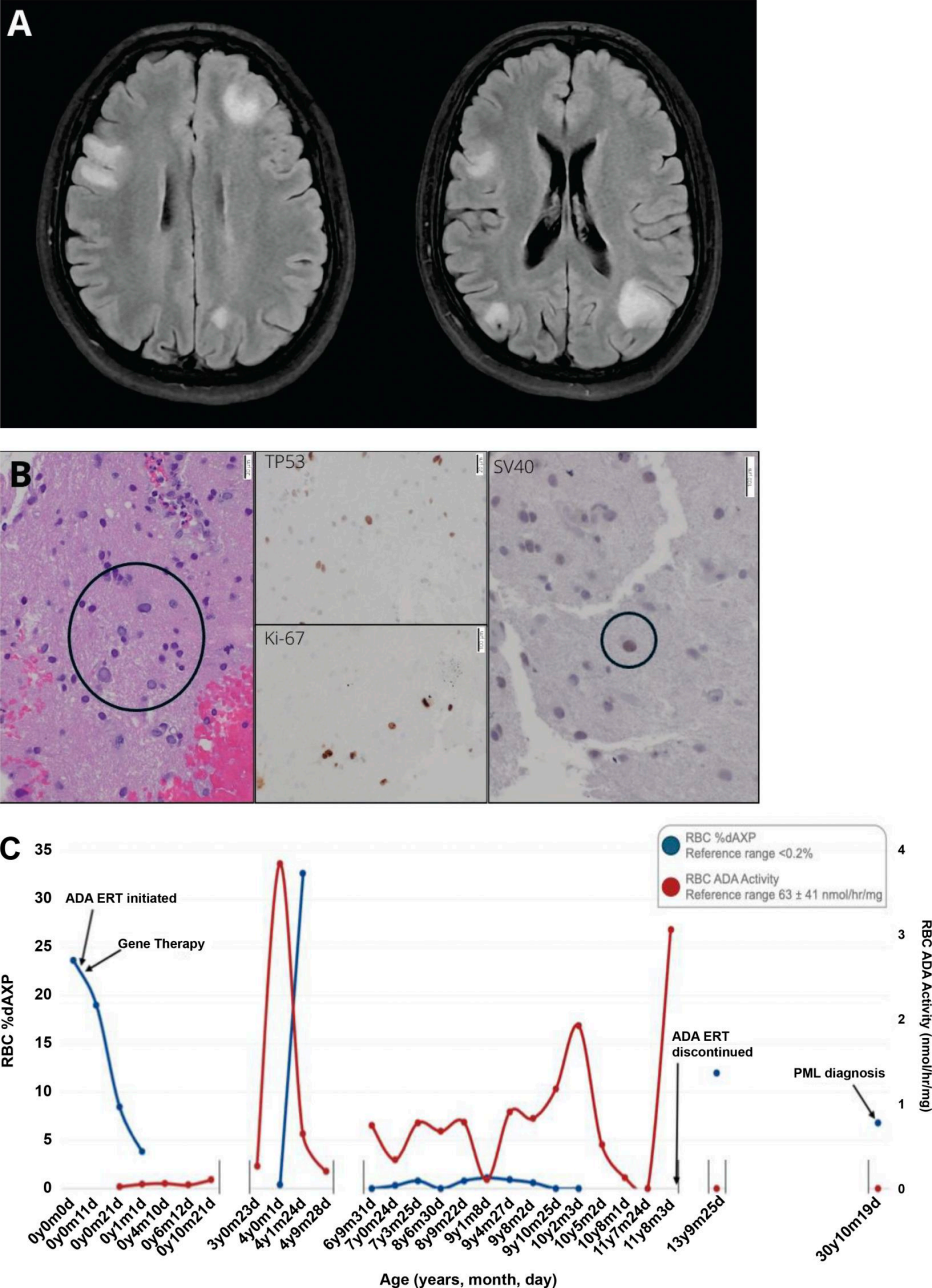
Brain MRI, brain biopsy, and longitudinal biochemical monitoring in this patient with ADA-SCID who developed progressive multifocal leukoencephalopathy.

A 31-year-old male presented to the emergency department (ED) with new-onset generalized seizures. He reported generalized weakness, cognitive slowing, and nausea for several weeks. He denied fatigue, weight loss, fever, dyspnea, abdominal pain, vomiting, diarrhea, arthritis, focal weakness, or slurred speech. Brain MRI demonstrated multiple hyperintense cortical and subcortical lesions (Fig. 1 A).

He was prenatally diagnosed and confirmed with severe combined immunodeficiency from adenosine deaminase deficiency (ADA-SCID) [c.95+1G>A; c.646G>A (p.Gly216Arg)] due to known maternal carrier status (1). Enzyme replacement therapy (ERT) was begun at 3 days of age (2). He underwent gene therapy (GT) via gammaretroviral (gRV)-vector-transduced autologous umbilical cord blood CD34^+^ stem cell transplant at 4 days of age without cytoreductive conditioning (2). Intravenous immunoglobulin was discontinued at age 4, and adherence to ERT was discontinued at age 12. He had poor compliance with medical care, including missed appointments and intermittent ERT, and was eventually lost to follow-up in adolescence.

Past medical history included autism spectrum disorder, strabismus, and abdominal dermatofibrosarcoma protuberans status post resection and local radiation without evidence of metastasis or recurrence. In childhood, he had multiple sinopulmonary infections requiring outpatient oral antibiotics, four episodes of varicella zoster requiring oral acyclovir, and one staphylococcal skin abscess requiring hospitalization. His last contact with immunology was documented in 2007; his mother reported since that time the patient had self-resolving viral upper respiratory infections yearly without other severe infections, hospitalizations, or immunosuppression.

Physical examination during his ED presentation at age 31 revealed a well-developed male with normal vital signs in no acute distress. Cardiopulmonary exam was normal, and abdominal exam did not reveal organomegaly. Neurologic exam was normal except for right eye exotropia, consistent with his childhood strabismus, and difficulty with two-step commands. Skin exam revealed no rashes or lesions.

Laboratory evaluation revealed a normal complete blood count with WBC 4.3 × 10^3^ cells/µl and absolute lymphocyte count 1,500 × 10^3^ cells/µl. Extensive infectious workup, including HIV testing, was unremarkable. Cerebrospinal fluid studies revealed undetectable human polyomavirus 2, also known as John Cunningham virus (JCV), viral load, unremarkable metagenomic analysis, no evidence of atypical or malignant cells, and no autoimmune encephalitis antibodies. Brain biopsy demonstrated hyperchromatic oligodendrocyte nuclei with ground-glass chromatin. Antigen kiel 67 and tumor protein p53 nuclear co-labeling was found as is characteristic of progressive multifocal leukoencephalopathy (PML), but simian vacuolating virus 40 immunohistochemistry showed only three lightly staining nuclei (Fig. 1 B). While not independently diagnostic, these results supported PML given the clinical and neuroimaging context.

Immunologic testing revealed normal CD3^+^ count (1,312 cells/µl [reference range 570–2,400]), normal CD4^+^ count (527 cells/µl [430–1,800]), normal CD8^+^ count (748 cells/µl [210–1,200]), low CD4:CD8 ratio (0.71 [1–4]), low CD19^+^ count (25 cells/µl [91–610]), and normal CD16^+^/CD56^+^ count (101 cells/µl [78–470]). Immunoglobulin levels were within normal range (IgG 1,229 mg/dl [700–1,600], IgA 152 mg/dl [70–400], and IgM 114 mg/dl [40–230]). Pneumococcal titers demonstrated 2 of 23 serotypes above 1.3 ug/ml in the setting of childhood vaccination, and tetanus titer was protective (0.5 IU/ml [>0.1]). Further testing demonstrated low absolute and percentage of CD45RA^+^ (56 cells/μl [150–870] and 11% [28–71], respectively) and normal absolute but elevated percentage of CD45RO^+^ (436 cells/µl [190–1,050] and 89% [28–72], respectively). Functional testing demonstrated undetectable red blood cell ADA activity (0 nmol/h/mg) and elevated percentage of deoxyadenosine (dAdo) nucleotide (%dAXP) (6.8% [<0.2], compared with 23.7% pre-ERT) (Fig. 1 C).

The patient was initially stabilized with levetiracetam and lacosamide; pembrolizumab was also started for PML (3). He was discharged with plan to restart ERT but was soon readmitted for worsening seizures; repeat imaging demonstrated disease progression. He developed refractory seizures despite multiple anti-epileptic medications. The family ultimately chose comfort care, and he died shortly thereafter.

## Discussion

This patient was diagnosed with PML based on clinical and radiologic findings, presence of histopathologic surrogate markers, and lack of more plausible explanations. PML primarily occurs in immunocompromised individuals, with known cases associated with advanced HIV, hematologic malignancy, transplant, primary immune deficiency, and immunosuppressive medications (3). The immunologic mechanisms of PML pathogenesis remain incompletely understood, but additional factors beyond prolonged cellular immune impairment must contribute since PML remains rare even within these immunocompromised populations (3). In vitro models have demonstrated JCV reactivation involves the nuclear factor κ-light-chain-enhancer of activated B cells pathway (3), a key signaling pathway regulating inflammation. B cells have also been proposed to serve as viral reservoirs, modulate T cell responses, and regulate cytokine secretion (3). Spi-B, a transcription factor involved in B cell differentiation and maturation, has also been found to regulate JCV gene expression (3).

One 39-year-old patient with ADA-SCID has been previously described to have PML (4). She experienced severe recurrent infections and lymphopenia in childhood before ADA deficiency was known. She survived and was relatively stable until adulthood, when she developed severe sinopulmonary infections from humoral immunodeficiency, elevated IgE, and hepatobiliary disease. Metabolic studies confirmed a diagnosis of ADA deficiency (4). She died from neurologic complications shortly after PML diagnosis (4). Her cellular and humoral immune dysfunction, like this patient, progressively deteriorated until reaching critical levels in adulthood.

Our leading hypothesis is the patient had inadequately sustained engraftment leading to dAdo toxicity and lymphocyte dysfunction. The patient’s labs demonstrated low CD4^+^CD45RA^+^ counts, low ADA activity, and elevated %dAXP, suggesting a persistent enzymatic defect. Prior data demonstrated absent T cell response to mitogen stimulation (Table 1). Definitive gene engraftment data were unable to be collected at the time of presentation and could not be retrieved 30 years after the original transduction, limiting further conclusions with the significant lapse in data.

**Table 1.** Timeline of labs from 1997 until time of presentation in 2024 (mm/dd/yy), including absolute lymphocyte count, CD3, CD19, CD16/56, CD45RO, and mitogen response

​	5/19/97 (ERT)	6/13/97 (no ERT)	3/14/00 (low-dose ERT)	9/6/00 (low-dose ERT)	12/15/00 (low-dose ERT)	6/5/01	10/9/02	4/8/03	10/14/03	1/14/04	1/6/05	3/17/05	4/6/05	8/25/05 (no ERT)	3/15/07	3/30/07 (no ERT)	6/18/07 (no ERT)	2/23/24 (no ERT)
ALC (cells/µl)	​	​	​	​	​	​	​	​	​	​	​	​	​	​	​	2.2	1.6	4.3
CD3^+^ (cells/µl)	​	154	​	460	​	499	319	653	731	365	868	1,325	2,623	1,609	1,819	​	1,429	1,500
CD19^+^ (cells/µl)	​	​	​	152	​	102	50	34	38	30	4	11	86	36	2	​	3	25
CD16^+^56^+^ (cells/µl)	​	​	​	​	​	​	​	​	​	​	3	19	​	22	3	​	10	101
CD45RO^+^ (cells/µl)	93	​	​	​	​	​	​	​	​	​	​	​	​	​	​	​	829	436
Mitogen response	​	​	Low response to PHA.	Low response to PHA.	Normal response to PHA.	Normal response to PHA.	Low response to PHA.	Low response to PHA.	Low response to PHA.	Normal response to PHA.	Low response to PHA.	Low response to PHA.	Low response to PHA.	Normal response to PHA.	​	​	Normal response to conA, PHA, and PWM.	​

Some clinical notes were available to verify whether the patient was on ERT, outgrowing his dose of ERT (low-dose ERT), or not on ERT (no ERT). ALC, absolute lymphocyte count. ConA, concanavalin A. PHA, phytohemagglutinin. PWM, poke weed mitogen.

Several factors likely contributed to poor gene engraftment, including low number of CD34^+^ gene-corrected cells, prolonged post-transduction use of polyethylene glycol-modified ADA, which reduced the selective advantage for gene-corrected cells, and lack of cytoreductive conditioning to prepare the marrow for engraftment (5). Early autologous GT protocols without preconditioning demonstrated partial but incomplete immune reconstitution (2, 5). This patient and two other ADA-deficient infants received autologous umbilical cord blood CD34^+^-cell transplant and maintained normal dAXP levels after reducing ERT, suggesting some transferred metabolic function (2). However, none were able to discontinue ERT. This patient was trialed off ERT but developed sinusitis, lymphopenia, and lost previously protective tetanus titers (2). Multiple providers also noted ERT nonadherence and missed medical appointments, which may explain his fluctuating ADA activity throughout childhood (Fig. 1 C).

Aside from his CD19^+^ count, his lymphocyte counts were within normal limits, and his tetanus titer was protective at time of presentation. His mother reported the patient fared clinically well and his %dAXP remained below pre-GT levels despite no ADA-SCID–related treatment for almost 20 years after GT. Prior data demonstrated normal CD3^+^ counts and normal response to concanavalin A (conA) and phytohemagglutinin (PHA) mitogens in 2007 when clinical documentation confirmed the patient was off ERT for at least 1 year (Table 1), suggestive of some cellular immunity. Minor engraftment may have stabilized within a subset of T and natural killer cell progenitors, resulting in a lineage-restricted form of mixed chimerism. His elevated percentage of CD4^+^CD45RO^+^ also led to speculation of dysfunctional, oligoclonal T cell expansion, which may explain some of the above findings. Further proliferation testing, gene marking, and TCR v-βrepertoire spectratyping analysis could not be performed prior to the patient’s death but would have been useful to better characterize the specific defect.

Subsequent protocols with pretransplant cytoreductive conditioning and higher doses of transduced CD34^+^ cells have improved ADA-SCID GT outcomes (5). gRV vectors were initially used for transduction given their ability to integrate proviral DNA into hematopoietic stem cells via reverse transcription. Lentiviral (LV) vectors containing cellular promoters have shown more efficient transduction and a better safety profile due to their larger carrying capacity, ability to integrate proviral DNA without mitosis, and use of self-inactivating vectors to reduce the risk of enhancer-mediated insertional mutagenesis (5). ADA-SCID gRV-GT is currently approved in Europe, and LV-GT is under clinical investigation in Europe and the United States with excellent long-term reconstitution of immunity (5). As exemplified in our patient’s case, long-term monitoring is essential to understand the dynamics of immune reconstitution and engraftment durability as GT protocols continue to be explored.

Interestingly, patients with ADA deficiency often exhibit neurologic and behavioral abnormalities, including cognitive impairment, seizures, attention deficit disorder, sensorineural hearing loss, and MRI abnormalities (6). These findings persist after hematopoietic stem cell transplant and ERT (6). One study demonstrated ADA-deficient mice exhibited reduced exploration and anxiety-like behavior that did not significantly correct after ERT (6). These findings corresponded with reduced brain volume, white matter alterations, and elevated total brain concentrations of Ado compared to wild type (6). This study did not investigate the role of dAdo, the principal mediator of lymphocyte toxicity in ADA-SCID. Better understanding the effects of specific metabolites on central nervous system function, such as glial antiviral response and blood–brain barrier permeability, may help uncover novel therapeutic targets for disorders along the neuro-immune axis.

## Ethics and informed consent

Oral informed consent was obtained from the patient and guardian for publication of this case report, which was exempt from IRB approval (1).
